# STAT1 is a key gene in a gene regulatory network related to immune phenotypes in bladder cancer: An integrative analysis of multi‐omics data

**DOI:** 10.1111/jcmm.16395

**Published:** 2021-02-19

**Authors:** Hong Weng, Shuai Yuan, Qiao Huang, Xian‐Tao Zeng, Xing‐Huan Wang

**Affiliations:** ^1^ Department of Urology Zhongnan Hospital of Wuhan University Wuhan China; ^2^ Center for Evidence‐Based and Translational Medicine Zhongnan Hospital of Wuhan University Wuhan China; ^3^ Center for Evidence‐Based and Translational Medicine Wuhan University Wuhan China; ^4^ Institute of Urology Wuhan University Wuhan China

**Keywords:** bladder cancer, GEO, immunophenotype, prognosis, STAT1, TCGA

## Abstract

The immunophenotype of bladder cancer plays a pivotal role in the prognosis of cancer, but the effect of different epigenetic factors on different immunophenotypes in bladder tumours remains unclear. This study used multi‐omics data analysis to provide molecular basis support for different immune phenotypes. Unsupervised cluster analysis revealed distinct subclusters with higher (subcluster B2) or lower cytotoxic immune phenotypes (subcluster A1) related to PD‐L1 and IFNG expression. Mutational landscape analyses showed that the mutation level of TP53 in subcluster B1 was highest than other subclusters, and subcluster B1 had a lower frequency of concurrent mutation than subcluster A2. A total of 2364 differentially expressed genes were identified between subclusters A2 and B1, and the main functions of the up‐regulated genes in subcluster B1 were enriched in the activation of T cells and other related pathways. We found that STAT1 was a key gene in a gene regulatory network related to immune phenotypes in bladder cancer. Finally, we constructed a prognostic prediction model by LASSO Cox regression which could distinguish high‐risk and low‐risk cases significantly. In conclusion, the present study addressed a field synopsis between genetic and epigenetic events in immune phenotypes of bladder cancer.

## 1 INTRODUCTION

Bladder cancer is ranked tenth in all cancer worldwide, fourth in American men and seventh in Chinese men.^1^, ^2^, ^3^ The incidence of bladder cancer is related to gender, in which the incidence of the disease in men is significantly higher than that in women. This type of cancer can occur at any age. The vast majority of bladder cancer patients are non‐muscle‐invasive disease (more than 70%).^4^ The non‐muscle‐invasive bladder cancer patients are usually diagnosed and treated with transurethral resection of bladder tumours and can be followed by intravesical chemotherapy or immunotherapy. However, the recurrence rate of bladder cancer can be more than 70%.^5^ Besides, the high risk of no‐muscle‐invasive disease can develop into muscle‐invasive cancer. The disease burden of bladder cancer is very high worldwide, and the mechanisms of aetiology and progression of cancer remain unclear. Therefore, to study the mechanism of bladder cancer tumorigenesis and progression is urgent.

Recently, immune checkpoint inhibitors targeted on anti‐PD‐1 antibodies have dramatically changed the therapeutic landscape of patient with multiple carcinomas, including bladder cancer.^6^, ^7^, ^8^, ^9^, ^10^, ^11^, ^12^ However, not all bladder cancer patients can benefit from immune checkpoint inhibitors with anti‐PD‐1 antibodies. Researchers suggest that the tumour microenvironment is infiltrated with many types of innate and adaptive immune cells, and the patients with pre‐existing T cell infiltration and PD‐L1 expression can be more likely to benefit from the immune checkpoint inhibitors targeted on anti‐PD‐1 antibodies.^13^, ^14^ More recently, four immune subtypes in bladder cancer were established.^15^ However, the underlying molecular principles of the tumour immune phenotypes have not been exhibited detailedly and the prognostic prediction model has not been constructed.

Therefore, the present study reused multi‐omics data obtained from public databases, including The Cancer Genome Atlas (TCGA) and Gene Expression Omnibus (GEO), to uncover the influence of genetic and epigenetic alterations on different immune phenotypes in bladder cancer. Also, we identified key a gene in gene regulatory networks related to immune phenotypes in bladder cancer and constructed a prognostic prediction model for bladder cancer patients.

## 2 MATERIALS AND METHODS

### 2.1 Data acquisition

Expression of mRNA, miRNA and lncRNA, phenotype data and survival data were downloaded from UCSC Xena (https://xenabrowser.net) in July 2020. A total of 430 cases (including 19 normal bladder tissues) were obtained from TCGA‐BLCA cohort. Transcriptome data with more than 80 cases for validation cohorts were obtained from GEO (https://www.ncbi.nlm.nih.gov/geo/) in July 2020. A total of five GEO data sets were identified, GSE87304 ^16^ (305 bladder cancers), GSE128702 ^17^ (256 bladder cancers), GSE31684 ^18^ (93 bladder cancers), GSE13507 ^19^ (165 bladder cancers) and GSE154261 ^20^ (99 bladder cancers). The clinical information of included bladder cancers was presented in Table S1.

### 2.2 Immune infiltration analysis and unsupervised cluster analysis

The processed transcriptome data were upload to CIBERSORTx (https://cibersortx.stanford.edu) for performing immune infiltration analysis.^21^ The data of 22 types of immune cells were downloaded. Then, the expression data of PD‐L1 and IFNG were used to screen for immune cell types that are highly correlated with their expression levels. We used eight most correlated immune cells combined with Lasso regression to construct prediction models of PD‐L1 and IFNG expression levels, to find out the immune cells that are highly correlated with their expression levels. We used the immune cell types (T cell CD8, T cells CD4 memory activated and macrophages M1) that were simultaneously highly correlated with the expression of PD‐L1 and IFNG and that Lasso regression analysis showed that their expression levels were more predictable for unsupervised cluster analysis (dist = Euclidean, method = ward.D2). The unsupervised cluster analysis was performed to classify bladder cancer patients into different groups.

### 2.3 Somatic mutation analysis

The somatic mutation data were obtained from UCSC Xena and TCGA official website (https://portal.gdc.cancer.gov) and analysed with VarScan2.^22^ The somatic mutation data of each group were extracted using R software. We used ‘maftools’ (version 2.4.05) package for gene mutation analysis, mutual exclusion analysis and collaboration analysis.

### 2.4 Differentially expressed gene analysis and functional enrichment analysis

The differential gene analyses of mRNA, miRNA and lncRNA (data were obtained from UCSC Xena) were performed using the ‘DESeq’ package (version 3.11) in R (*P*.adj < 0.05 and |logFC| > 1), and the volcano plot is plotted using the plot function in R (R version 3.6.3). To study the functional enrichment regions of the genes up/down‐regulated in each group of differential genes, the ‘clusterProfiler’ (version 3.14.3) package was used for gene enrichment analysis.

### 2.5 DNA methylation analysis

The TCGA DNA methylation data were obtained from UCSC Xena. The ‘impute’ (version 3.11) package was used to fill in the missing values using the k‐nearest neighbour (kNN) algorithm, and ‘minfi’ (version:3.11) package was used to analysis the differential probes (FDR < 0.001 and |logFC| > 0.05). The platform annotation file in NCBI was used to map each probe to the genes one‐to‐one, and then, we got the intersection between the corresponding genes and the differentially expressed genes.

### 2.6 Copy number variation analysis

To study the difference in gene copy number between different groups, the copy number data of the genome fragment was obtained from UCSC Xena, and then, the information of the genome fragment corresponded with the position information of the genes, and the differences in the copy number of the genes were extracted. Then, we used the chi‐square test in R to analyse non‐copy number variation patients and samples with abnormal copy numbers in different groups (*P* < 0.05). Visualization analysis was performed using the Integrative Genomics Viewer (IGV) software (version 2.8.3) based on the copy number difference and their corresponding location information.^23^ The intersection between differentially expressed genes and genes with significant differences in copy number was generated.

### 2.7 Target gene prediction of miRNA and lncRNA

We first used the highly conservative prediction network in the miRcode^24^ (version miRcode11, http://www.mircode.org/) database to predict the miRNA target genes regulated by lncRNA and used the target genes to intersect the differentially expressed miRNA. Target genes prediction for differentially expressed miRNA and miRNA predicted by differentially expressed lncRNA was performed using TargetScan^25^ (version 6.0 for miRcode11, http://www.targetscan.org/vert_60/; version 7.2 for differentially expressed miRNA, http://www.targetscan.org/vert_72/), miRDB^26^ (version 6.0, http://mirdb.org) and miRTarBase^27^ (version 8.0, http://mirtarbase.cuhk.edu.cn/php/index.php). The target genes predicted by each database were analysed and take the intersection between the results of each database by Venn plot. The targeted network predicted by at least two databases at the same time was used as a significant regulatory network.

### 2.8 Construction of miRNA‐lncRNA‐mRNA regulatory network and hub gene identification

We used the obtained lncRNA‐miRNA, the mRNA in the miRNA‐mRNA regulatory network and the String database to construct the lncRNA‐miRNA‐mRNA interaction network. The interaction network was visualized using Cytoscape (version 3.8.0). To find the core gene in the interaction network, the MCC algorithm in the cytoHubba plugin was used to extract the hub gene.^28^ The effect of the hub gene in bladder cancer patients was further explored using the Oncomine database (https://www.oncomine.org/resource/login.html).

To study the influence of DNA methylation and copy number on hub gene expression, we checked whether the DNA methylation probes related to the hub gene have significant differences between different immunophenotypes. At the same time, we checked whether the region with a significant difference in DNA copy number overlaps with the hub gene. Besides, we used SMART (http://www.bioinfo‐zs.com/smartapp/) to analyse the relationship between DNA methylation of hub gene and patients’ survival.^29^

To study the relationship between the hub gene and the immune status of bladder cancer patients, correlation analysis between the proportion of immune cells in each sample obtained by CIBERSORTx and the expression of the STAT1 gene was performed.

### 2.9 Lasso cox regression model construction and validation

To find the genes related to prognosis among the differentially expressed genes and construct a prognostic model to provide indications for clinical treatment, we constructed a Lasso Cox regression model. First, we used the differentially expressed genes between different immunophenotypes to analyse the significance of single‐gene prognosis (*P* < 0.001). Subsequently, Cox regression analysis was performed using a single‐gene prognostic analysis of significant genes prognosis combined with survival information. To further optimize the model, we combined with the Lasso regression model to build a multi‐factor prognostic prediction model using the level of risk to divide patients into high‐risk and low‐risk groups and analysed the prognosis of the two groups to prove the effectiveness of the model. To verify the effectiveness of the model, additional data (GSE31684) were used to validate the constructed model based on TCGA‐BLCA. The ‘survminer’, ‘survival’ and ‘ggplot’ packages were used for survival analysis.

## 3 RESULTS

### 3.1 Classification of immunophenotypes in bladder cancer

**FIGURE 1:**
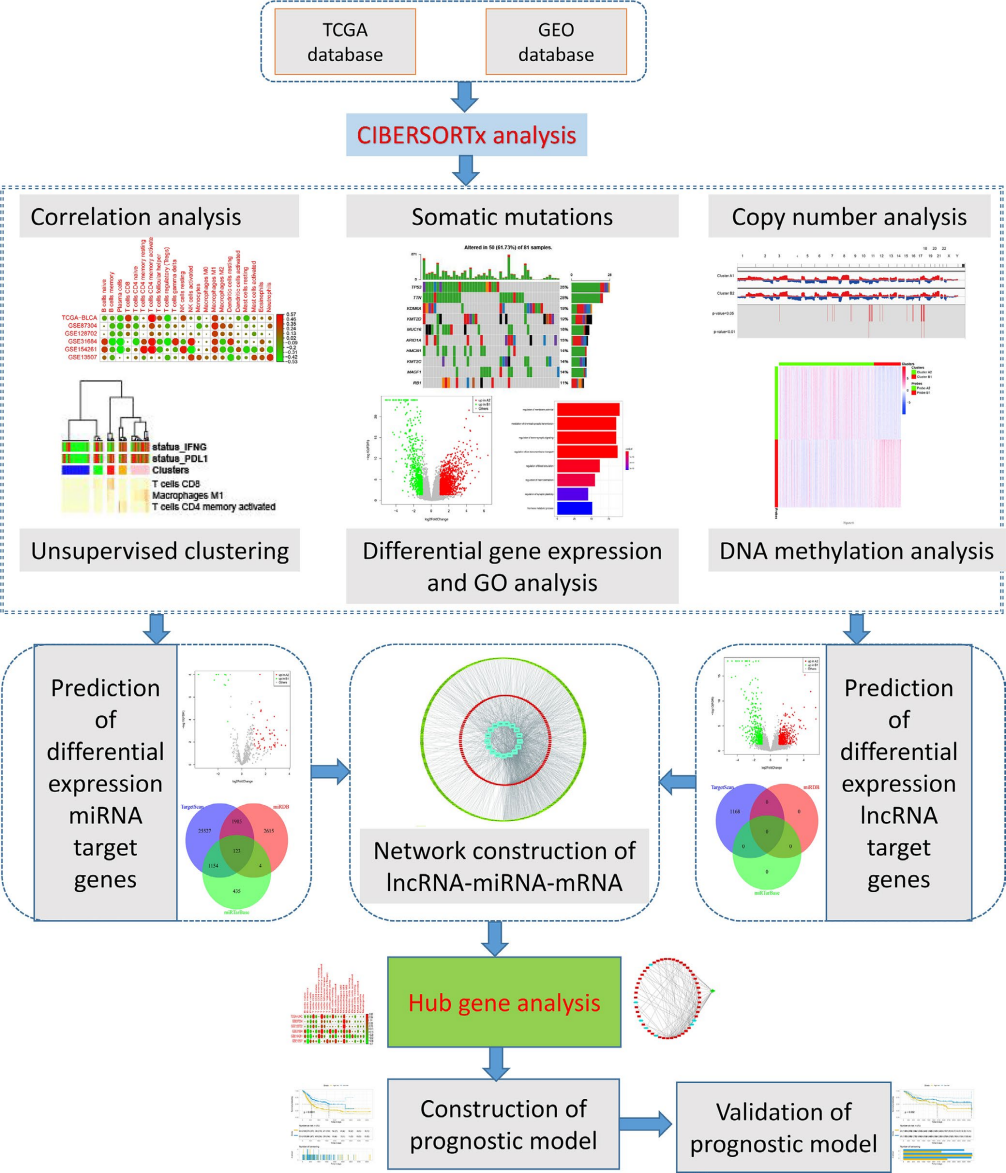
Flowchart of integrative analysis of multi‐omics data in bladder cancer in the present study. GEO, Gene Expression Omnibus database; GO, Gene Ontology; TCGA, The Cancer Genome Atlas

**FIGURE 2:**
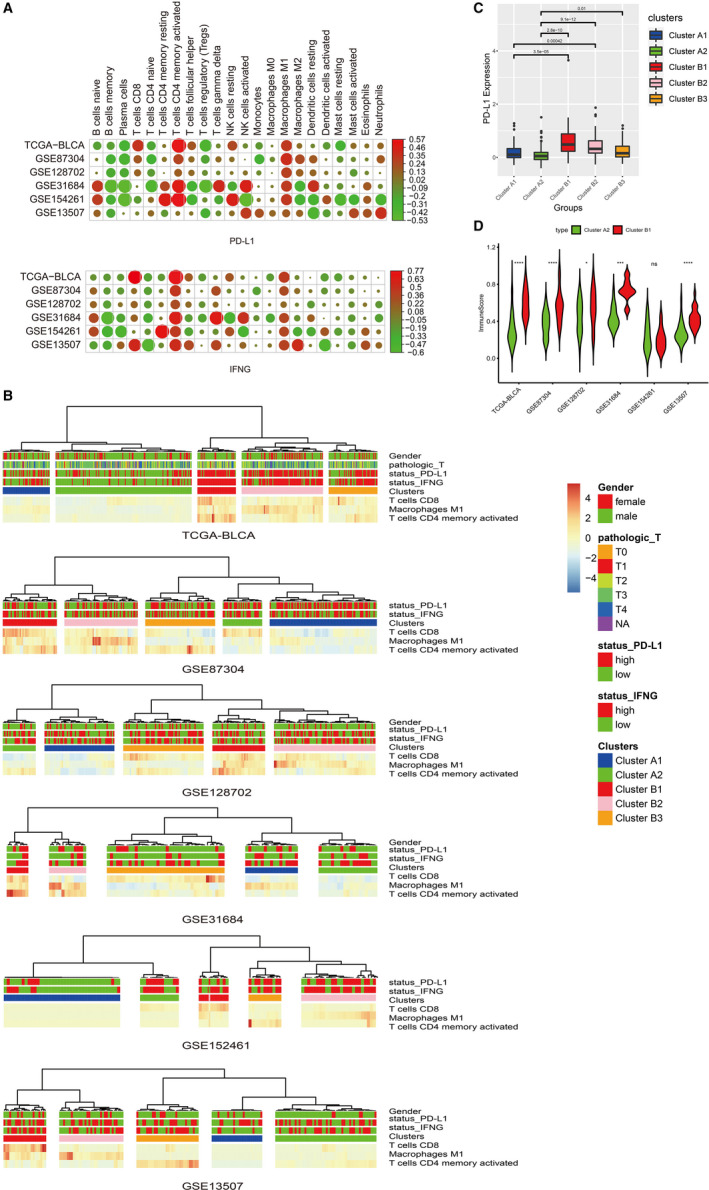
A novel molecular immune classifier of five subclusters by LASSO regression. A, Correlation analysis between either PD‐L1 or IFNG transcript levels and 22 immune cell phenotypes in TCGA‐BLCA and five GEO data sets. B, Unsupervised hierarchical cluster analysis. C, The difference in PD‐L1 protein expression levels between subclusters. D, The difference in immuneScore between subclusters. * *P* < 0.05; *** *P* < 0.001; **** *P* < 0.0001; ns, no significance

Six independent transcriptome data sets including 1329 bladder cancer patients, including TCGA‐BLCA (n = 411), GSE87304 ^16^ (n = 305), GSE128702 ^17^ (n = 256), GSE31684 ^18^ (n = 93), GSE13507 ^19^ (n = 165) and GSE154261 ^20^ (n = 99) were analysed by CIBERSORTx to evaluate portion of 22 immune cell types in bladder cancer patients. The analysis process was presented in Figure 1. The TCGA‐BLCA data set was a training cohort and GSE data sets were validation cohorts. Spearman´s correlation analysis and Lasso regression analysis showed that T cell CD8, T cells CD4 memory activated and Macrophages M1 immune cell subtypes were positive correlation between PD‐L1 and IFNG transcript levels and individual immune cell scores (Figure 2A).

An unsupervised hierarchical cluster analysis of the TCGA‐BLCA cohort based on the abovementioned three immune cell subtypes revealed two main clusters A and B (Figure 2B). Patients in cluster B had higher infiltration by all three abovementioned immune cell subtypes than cluster A. Cluster A was classified into two subclusters A1 and A2. In cluster A, subcluster A1 had higher infiltration than subcluster A2. Cluster B was classified into three subclusters B1, B2 and B3. In cluster B, subcluster B1 was presented with high infiltration by T cell CD8 and T cells CD4 memory activated. Similar patterns were observed by other GEO data sets. We also observed that patients in cluster B had a higher expression level of PD‐L1 than cluster A (Figure 2C). The PD‐L1 expression level was gradually decreased with the decrease of immune activity in subclusters B1, B2 and B3. Therefore, we identified A2 and B1 subclusters as the research objects to study the difference between low cytotoxic immune phenotype (A2) and high cytotoxic immune phenotype (B1) in bladder cancer patients. In the TCGA‐BLCA cohort, the ssGSEA enrichment score of expanded immune genes (immuneScore) was significantly higher in subclusters B1 than A2. Similar patterns were found in GSE87304, GSE128702, GSE31684 and GSE13507, except for GSE154261 (Figure 2D).

### 3.2 Somatic mutation difference in bladder cancer patients related to different immune phenotypes

**FIGURE 3:**
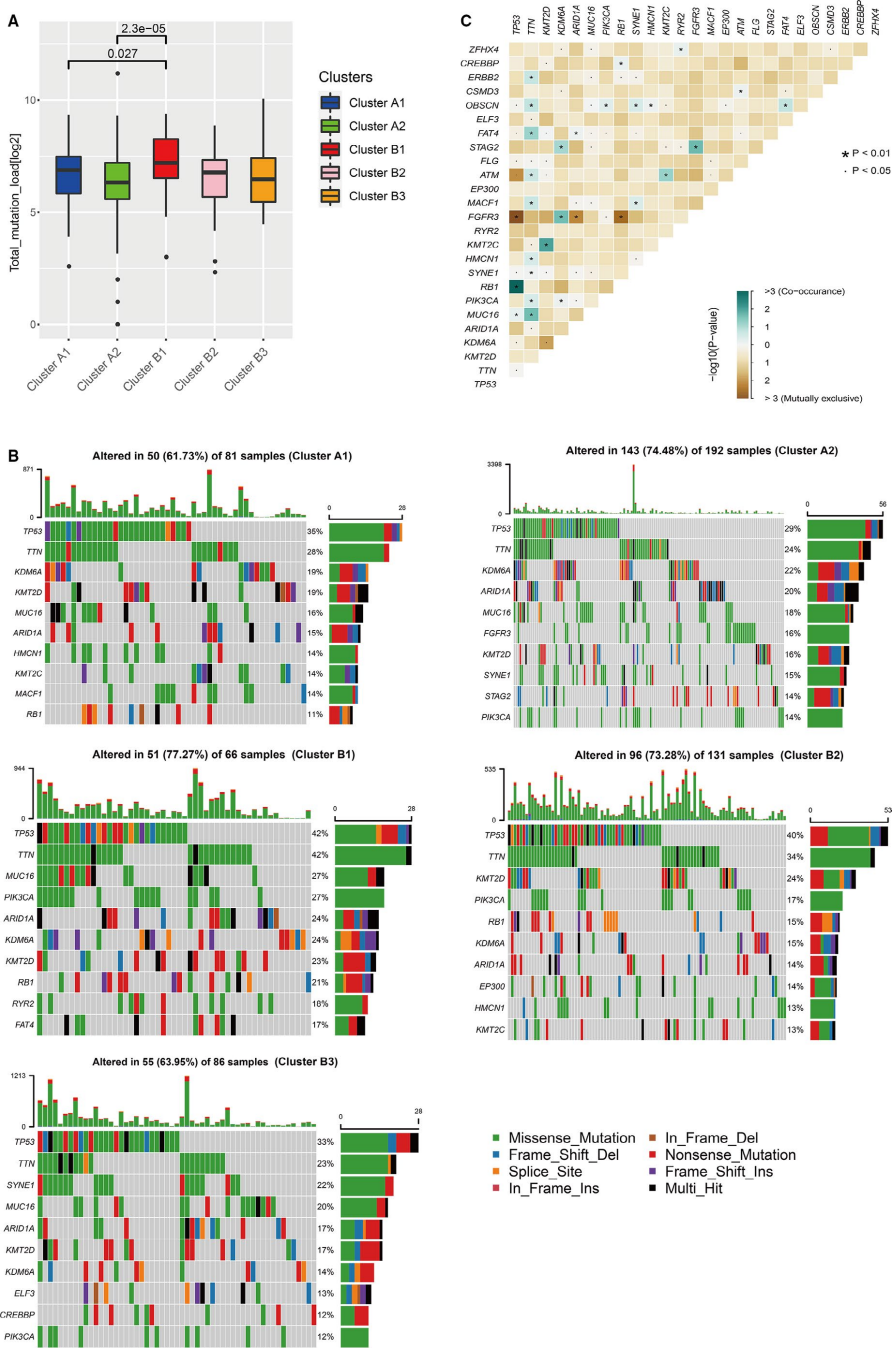
Somatic mutation difference in bladder cancer patients related to different immune Phenotypes. A, The overall status of somatic mutations in different groups. B, Gene mutation frequencies in different subclusters. C, The analyses of concurrent and mutually exclusive mutations among the overall gene mutations in the TCGA‐BLCA patients

To study the differences in somatic mutations between different immune phenotypes, the somatic mutation data in TCGA were used to analyse the number and proportion of somatic mutations in each subcluster identified in the TCGA‐BLCA cohort. Analysis of the total amount of mutations between each subcluster showed that there were significant differences between subclusters A1, A2 and B1, among which subclusters A2 and B1 were more significant (Figure 3A). It indicated that the immune status of different subclusters might be related to somatic mutations. Then, we analysed the genes with higher mutation frequency in each subcluster. We observed that the mutation frequencies of TP53 and TTN were both high in cluster A and cluster B (Figure 3B). However, the mutation level of TP53 in subcluster B1 was highest than other subclusters. In cluster B, the mutation frequency of TP53 and TTN in subclusters B1, B2 and B3 presented a gradual decrease. In Cluster A, the mutation levels of TP53 and TTN in subcluster A1 were slightly higher than subcluster A2; however, the mutations levels of KDM6A, MUC16 and ARID1A in subcluster A1 were lower than subcluster A2.

The analyses of concurrent and mutually exclusive mutations among the overall gene mutations in the TCGA‐BLCA patients found that TP53 had concurrent mutations with RB1 and MUC16, and had mutually exclusive mutations with FGFR3, ATM and other genes (Figure 3C). We also found that the TTN gene had significant concurrent mutations with many genes, such as ERBB2, OBSCN, FAT4, ATM and KMT2C. Besides, to study the differences in the concurrent and mutually exclusive mutations between different subclusters, we carried out the concurrent and mutually exclusive mutations analysis of TCGA‐BLCA patients for each subcluster. The results showed that among the genes with significant mutually exclusive or concurrent mutations, the vast majority were the types with significant concurrent mutations. The resultes of concurrent mutation analyses between clusters/subclusters showed that more genes had concurrent mutations with TP53 in cluster A compared with cluster B, and subcluster B1 had a lower frequency of concurrent mutation than subclusters A1 and A2. Otherwise, the concurrent mutation frequency of TTN in subcluster B1 was higher than cluster A, especially relative to subcluster A1. The epigenetic‐related methyltransferase gene KMT2D had a more concurrent mutation in cluster B1 than other subclusters. But there was no gene had concurrent mutation with KMT2D in subcluster A1. In cluster A, there were more genes in subcluster A1 that had concurrent mutation with HMCN1 and BRCA2; however, there were more genes that had concurrent mutation with FAT4 and OBSCN in subcluster A2. But compared with cluster B, more genes had concurrent mutation with TP53 in the two subclusters A1 and A2. In cluster B, more genes had concurrent mutation with TTN; different from subclusters B2 and B3, more genes had concurrent mutation with KMT2D, FAT1 and ATM in subcluster B1 (Table S2).

### 3.3 Differentially expressed genes and functional enrichment analysis

**FIGURE 4:**
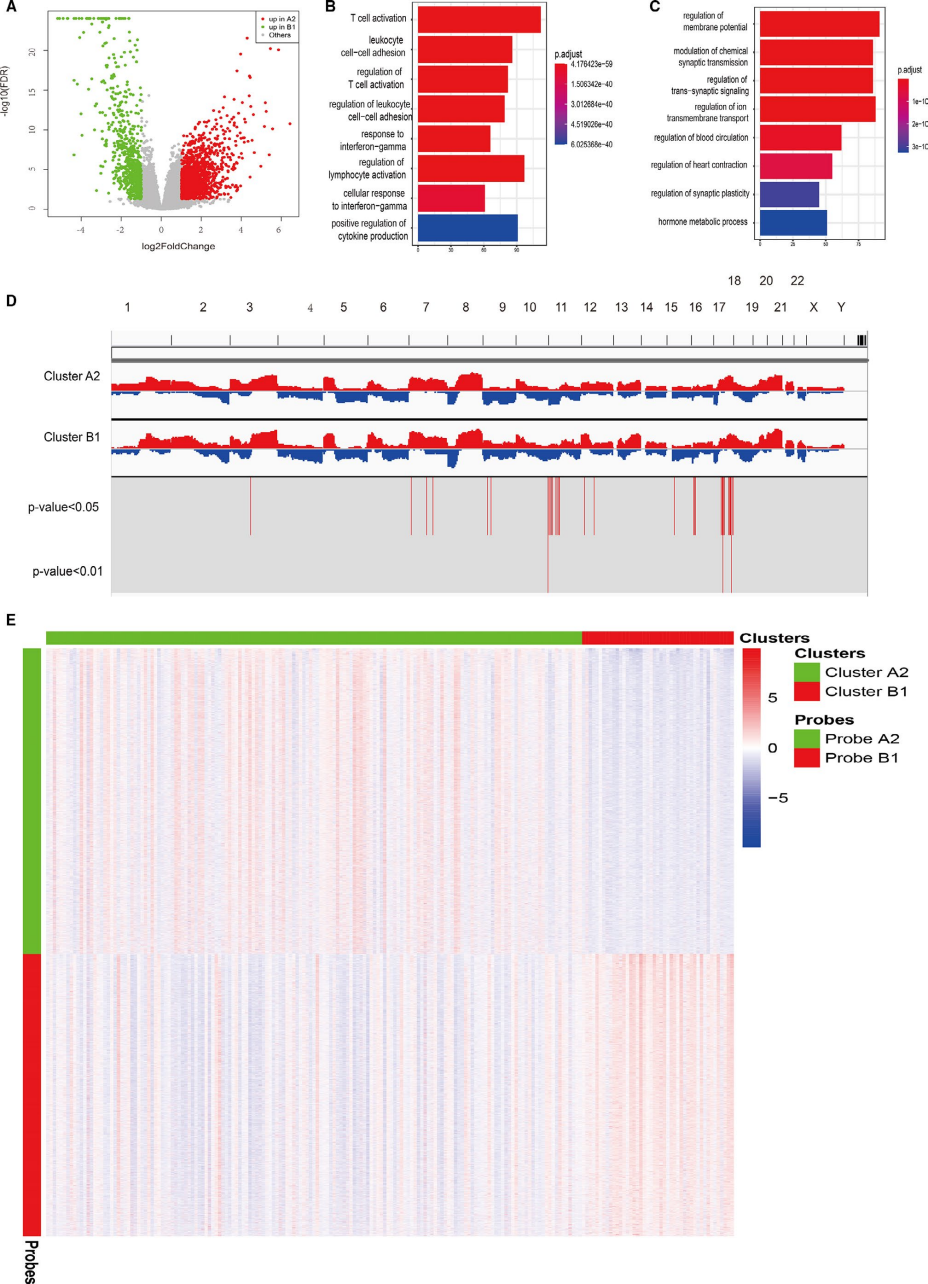
Differentially expressed gene, copy number variation and DNA methylation analysis between subcluster A2 and B1. A, Volcano plot of differentially expressed genes between subclusters A2 and B1. B, GO analysis of up‐regulated differential genes in subcluster B1. C, GO analysis of up‐regulated differential genes in subcluster A2. D, The analysis of gene copy number difference between subclusters A2 and B1. E, DNA methylation difference between subclusters A2 and B1. GO, Gene Ontology

A total of 2364 differentially expressed genes were identified between subclusters A2 and B1 (Figure 4A). Through GO function enrichment analysis, we found that the main functions of the up‐regulated genes in subcluster B1 were enriched in the activation of T cells and other related pathways, which is consistent with the status of high infiltration immune cells in this subcluster (Figure 4B). The functions of up‐regulated genes in subcluster A2 were mainly enriched in signal pathways related to excitation and nerve conduction (Figure 4C).

### 3.4 Differences in copy number variation related to immune phenotypes in bladder cancer

By comparing the copy number variation between subclusters A2 and B1, we found that there were 566 genes with increased copy number variations in subcluster A2, of which 47 of them were highly expressed in the mRNA level (*P* < 0.05). There were 24 genes with increased copy number variations in subcluster B1, of which two year of them were highly expressed in mRNA level (*P* < 0.05). Additionally, we found that the genes with increased copy number variations in subcluster A2 were mainly enriched on chromosomes 11 and 17, while the genes with increased copy number variations in subcluster B1 were mainly enriched on chromosome 16 (Figure 4D).

### 3.5 Differences in DNA methylation related to immune phenotypes in bladder cancer

To study the impact of changes in DNA methylation levels in different immune phenotypes, the DNA methylation data obtained in TCGA‐BLCA were used for the differential analysis of DNA methylation. By comparing the genes with different methylation levels between subclusters A2 and B1, we found that a total of 5831 probes were significantly up‐regulated in subcluster A2 (FDR < 0.001, |logFC| > 0.5) which corresponding to 5267 genes and a total of 5382 probes were significantly up‐regulated in subcluster B1 which corresponding to 3239 genes (Figure 4E). Among them, 222 genes corresponding to the probes up‐regulated in subcluster A2 appeared in the differentially expressed genes in subcluster B1. Otherwise, 276 genes corresponding to the probes were significantly up‐regulated in subcluster B1 appeared in the differentially expressed genes in subcluster A2.

### 3.6 Construction of miRNA‐lncRNA‐mRNA regulatory network related to immune phenotypes

**FIGURE 5:**
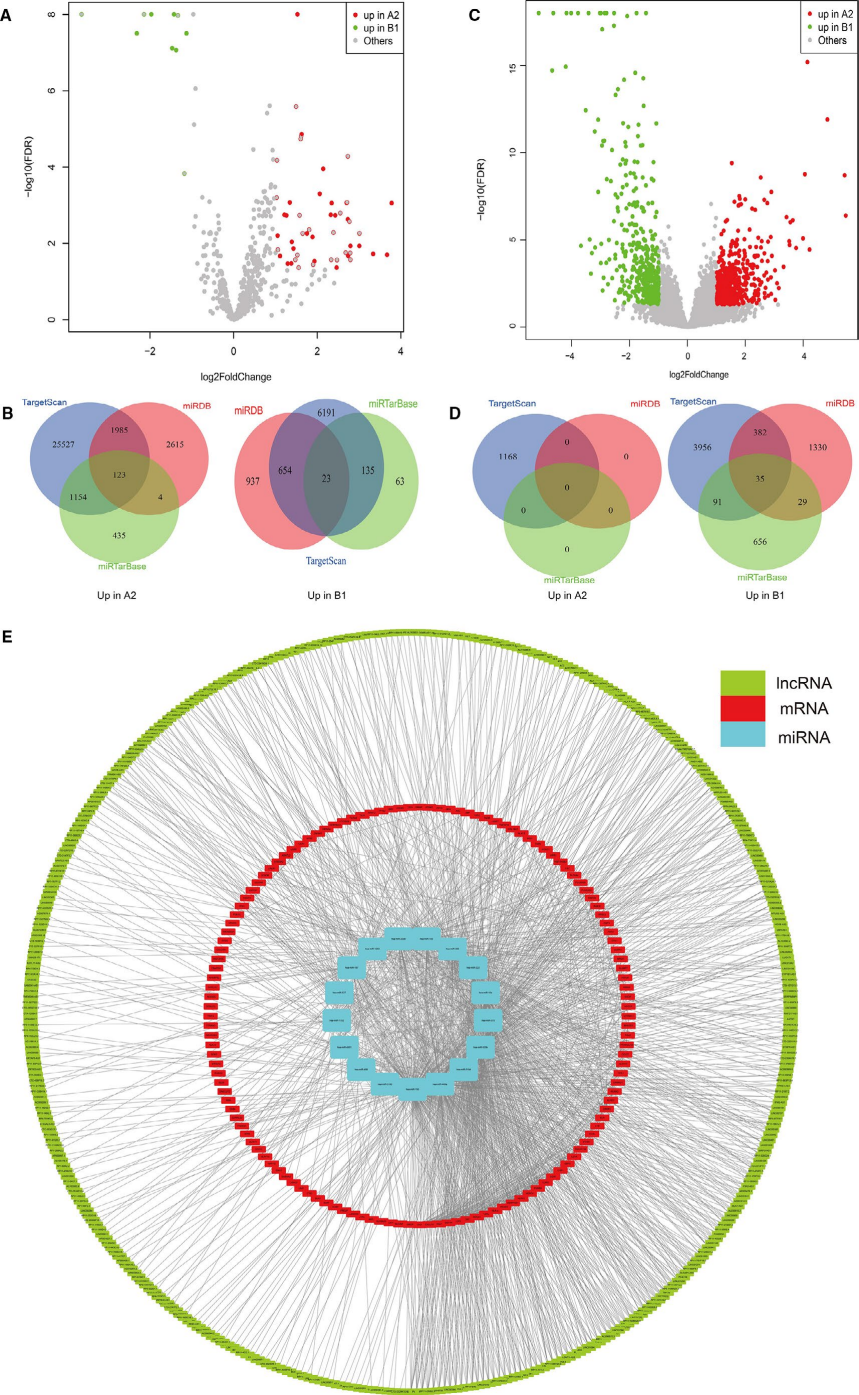
The miRNA‐lncRNA‐mRNA regulatory network construction. A, Volcano plot of differentially expressed miRNAs between subclusters A2 and B1. B, Venn plot for the prediction of miRNA target genes. C, Volcano plot of differentially expressed lncRNAs between subclusters A2 and B1. D, Venn plot for the prediction of miRNA target genes based on differentially expressed lncRNAs between subclusters A2 and B1. E, The miRNA‐lncRNA‐mRNA regulatory network

To assess the differential expression of miRNA in different immunophenotypes, we used the data of miRNA expression obtained in TCGA‐BLCA for differential analysis. The results of difference analysis between subclusters A2 and B1 found 62 differentially expressed miRNAs (FDR < 0.05), of which there were 52 significantly up‐regulated miRNAs in subcluster A2 and 10 in subcluster B1 (Figure 5A). These miRNAs were screened as candidate molecules, and databases (TargetScan, miRDB and miRTarBase) were used to predict target genes of candidate molecules. Target genes that could be predicted simultaneously in the two databases were identified as alternative regulatory networks. A total of 3266 predicted miRNA‐mRNA regulatory networks were found in the predicted network of miRNA regulation that was significantly up‐regulated in subcluster A2 and 812 were found in subcluster B1 (Figure 5B).

To study the differential expression of lncRNA in different immunophenotypes and the possible influence of the lncRNA‐miRNA interaction network on gene expression in different immunophenotypes, the data of lncRNA expression obtained in TCGA‐BLCA were used to carry out differential expression. The results of the difference analysis between subclusters A2 and B1 found a total of 930 differentially expressed lncRNAs (Figure 5C). Then, we used the differentially expressed lncRNAs to combine with the highly conserved miRcode database and the differentially expressed miRNAs to predict lncRNA‐miRNA interactions. The results of the prediction showed that a total of 537 miRNA‐mRNA regulatory networks up‐regulated in subcluster A2 and 0 in subcluster B1 (Figure 5D).

To study the regulatory network in subclusters A2 and B1, we used differentially expressed miRNA, mRNA and lncRNA combined with the String database to construct an interaction network (Figure 5E).

### 3.7 Hub gene identification

**FIGURE 6:**
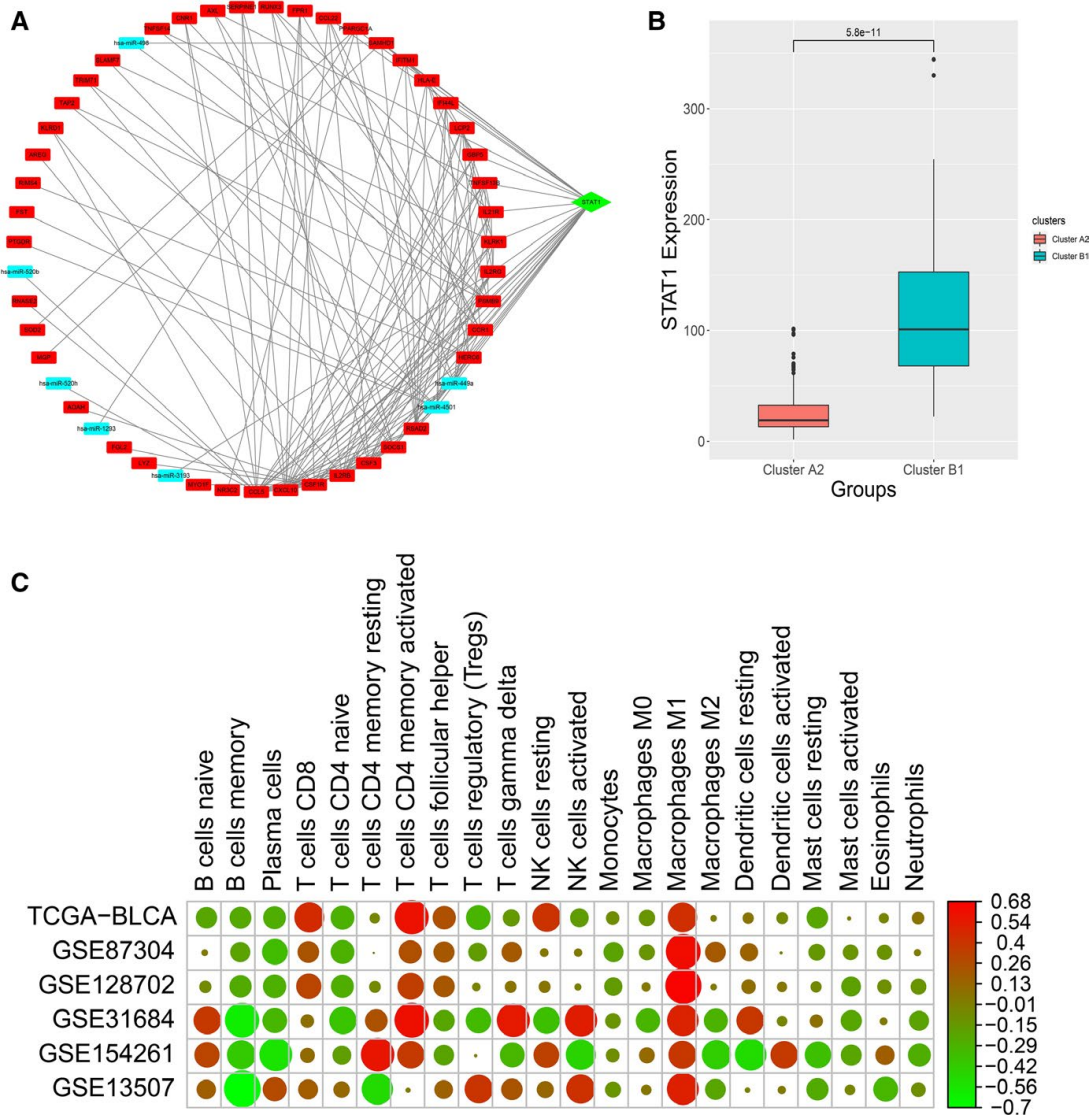
Hub gene analysis. A, Hub gene analysis and visualization. B, Hub gene expression level between subclusters A2 and B1. C, Correlation analysis between hub gene expression and 22 immune cell phenotypes in six bladder cancer data sets

To study the genes that might play a key role in the miRNA‐lncRNA‐mRNA regulatory network, we used the cytoHubba in Cytoscape to analyse the important hub genes in the network. Finally, we found that STAT1 ranked firstly in the network using the maximum correntropy criterion (MCC) algorithm. Therefore, STAT1 might play an important role in the differences in the immune conditions of cancer tissues. We extracted the STAT1‐related network and visualized it in Cytoscape (Figure 6A). Besides, we found that STA1 was significantly higher in subcluster B1 than subcluster A2 (Figure 6B).

To assess the influence of other epigenetic factors on STAT1, copy number variation data and DNA methylation data were analysed and we found that there was no difference in copy number of STAT1 gene between subclusters A1 and B2. However, the results of the differential analysis of DNA methylation indicated that the probe corresponding to the STAT1 gene was significantly up‐regulated in subclusters A2 and B1, which indicated that the DNA methylation level may affect the expression level of STAT1. We used Oncomine and SMART to validate the aforementioned results. We found that STAT1 was highly expressed in bladder cancer patients (Figure S1). Additionally, we found that six methylation probes (cg00137918, cg14951497, cg01085225, cg25856179, cg14768946 and cg15325732) were significantly different between bladder cancer and normal bladder (Figure S2). Spearman's analysis suggested that cg00137918, cg14951497, cg01085225, cg25856179, cg14768946, cg00493400, cg11556416 and cg15325732 were negatively correlated with STAT1 gene expression with statistical significance (Figure S3). Multivariate Cox regression analysis suggested that cg14951497 was associated with an increased risk of overall survival in bladder cancer patients (Table S3). No significant result was found for disease‐free survival (Table S4).

To study the relationship between the hub gene and the immune status of the samples, the proportion of immune cells in each sample obtained by CIBERSORTx and the expression of the STAT1 gene were used. The correlation analysis results showed that the expression of STAT1 was positively correlated with T cell CD8, T cell CD4 memory activated and Macrophages M1 (Figure 6C). However, there was a negative correlation with B cell memory, plasma cells and T cell naive. It indicated that STAT1 might regulate the immune state of the tissue or provide clues to the immune state. We then used the TIMER database to validate the results analysed by CIBERSORTx, and we found similar results (Figure S4).

### 3.8 Prognostic prediction model construction related to immune phenotypes

**FIGURE 7:**
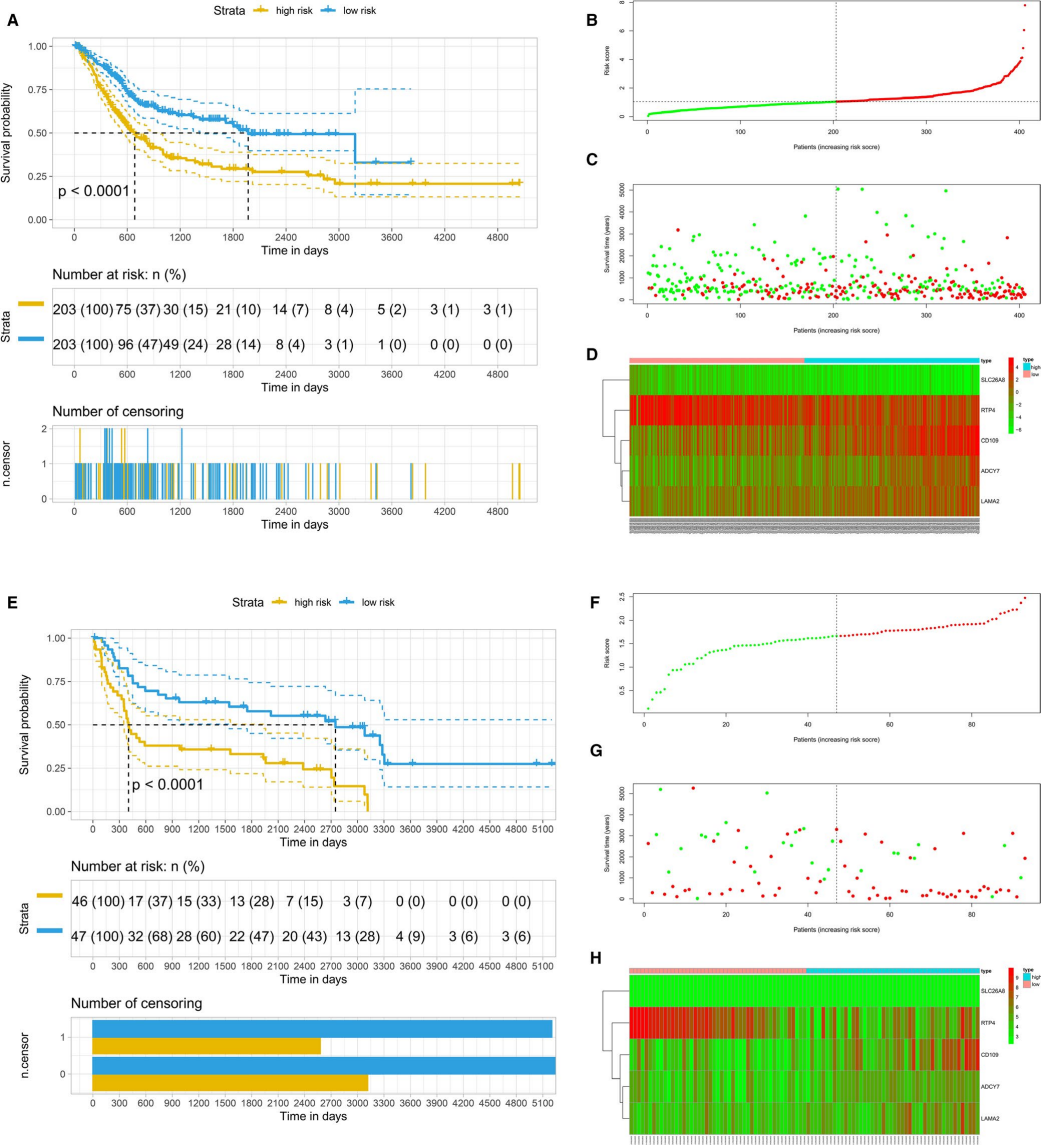
Construction and validation of the prognostic model based on LASSO Cox regressions. A, Kaplan‐Meier survival analysis of overall survival predictor based on five genes. B, Riskscore plot. C, Overall survival status and survival time of bladder cancer patients. D, The expression of five prognostic genes

A prognostic model based on differentially expressed genes between subclusters A2 and B1 was constructed, which could guide the clinical treatment of bladder cancer. A total of 54 significant prognostic genes correlated with bladder cancer overall survival were identified based on the differentially expressed genes between subclusters A2 and B1 using univariate Cox analysis. Then, Lasso Cox regression identified five relevant genes (coefficients of genes: ADCY7 = 0.11082592; LAMA2 = 0.0736452; RTP4 = −0.03958412; CD109 = 0.03188021; and SLC26A8 = −4.6472624). The constructed prognostic prediction model could distinguish high‐risk and low‐risk cases significantly (Figure 7A‐D). Also, we used an external data set (GSE31684) to validate the constructed model. We found that the model could significantly predict the prognosis of patients in GSE31684 data set as well (Figure 7E‐H). Moreover, the K‐M plots of genes involved in the prognostic prediction model based on TCGA‐BLCA data were presented in Figure S5.

## 4 DISCUSSION

Immunophenotype plays a pivotal role in the prognosis of bladder cancer. Therefore, a better understanding of the molecular basis of tumour microenvironment infiltrated with many types of innate and adaptive immune cells are urgent, which can improve the efficacy of the immune checkpoint inhibitors with anti‐PD‐1 antibodies in bladder cancer patients. In 2019, Cao et al^30^ using TCGA‐BLCA data set found four survival‐related immune cells and 24 hub genes, four of which were related to overall survival. Recently, there were many papers published using only TCGA‐BLCA data set to analyse the tumour‐infiltrating immune cells and prognostic genes related to the microenvironment of bladder cancer.^31^, ^32^, ^33^, ^34^, ^35^, ^36^, ^37^ Additionally, Song et al ^38^ using TCGA‐BLCA data set analysed immune‐related long non‐coding RNA signature for muscle‐invasive bladder cancer; Li et al^39^ using GES13507 data set analysed tumour microenvironment and found three immune‐related prognostic genes for bladder cancer; Luo et al^40^ and Qiu et al^41^ using TCGA‐BLCA as training cohort and GSE13507 as validation cohort constructed six and seven immune‐related genes prognostic signature, separately; Tian et al^42^ using TCGA‐BLCA cohort and four GEO data sets (GSE13507, GSE48075, GSE31684 and GSE32894) identified a prognostic signature based on six immune‐related genes. Tang et al^15^ using TCGA‐BLCA cohort and four GEO data sets (GSE13507, GSE32548, GSE31684 and GSE48276) identified four immune subtypes (referred to as C1‐C4) related to immune gene sets in bladder cancer and found that C2 was an immune‐infiltrating type and C4 was an immune ‘desert’ type. Only one study performed classification based on immune infiltrating cells, and three studies conducted validation for the prognostic prediction model. Moreover, the underlying epigenetic landscape between subtypes related immune phenotypes was not presented yet.

In the present study, we used TCGA‐BLCA cohort and five GEO data sets to performed immunophenotype classification, somatic mutation, copy number variation, DNA methylation, differentially expressed genes and corresponding functional enrichment analysis, lncRNA‐miRNA‐mRNA network, hub gene identification and prognostic prediction model construction and validation. We presented two main clusters (A and B) and five subclusters (A1, A2, B1, B2 and B3) for bladder cancer, in which subcluster B2 exhibited higher cytotoxic immune phenotype and subcluster A1 with lower cytotoxic immune phenotype. The two subclusters (A1 and B2) were identified for further analyses, such as mutational landscape and epigenetic patterns. We identified STAT1 as a key gene for tumour microenvironment related to immune cells, which could be further studied in vitro and in vivo for clinical treatment with immunotherapy. Otherwise, we also constructed a prognostic prediction model based on five immune‐related genes, including ADCY7, LAMA2, RTP4, CD109 and SLC26A8. These genes have not been identified in previous publications.

In somatic mutation analysis, we found that mutation levels of TP53 and TTN were both high in cluster A and cluster B. The analyses of concurrent and mutually exclusive mutations found that TP53 had concurrent mutations with RB1 and MUC16, and had mutually exclusive mutations with FGFR3 and ATM. The theory of ‘oncogene addiction’ may present a likely explanation for mutually exclusive mutations.^43^ The GO function enrichment analysis of differentially expressed genes between subclusters A2 and B1 found that the main functions of the up‐regulated genes in subcluster B1 were enriched in the activation of T cells. A previous study^44^ has suggested that FGFR3‐mutant bladder cancers are associated with decreased T cell infiltration, which supported our findings.

Several limitations existed in the present study. Firstly, the protein expression level of the hub gene lacked and its difference between subclusters related to different immune phenotypes could not be analysed. Secondly, no significant difference was found in copy number variation of the STAT1 gene between subclusters A1 and B2, which could not provide more evidence for the difference of STAT1 expression between immune phenotypes. Ultimately, our study is an in silico analysis reusing public data sets, which lack in vitro/in vivo functional validation studies.

## 5 CONCLUSION

In conclusion, the present study addressed a field synopsis between genetic and epigenetic events in immune phenotypes of bladder cancer, provided a significantly prognostic prediction model for bladder cancer and found that STAT1 was a key gene in a gene regulatory network related to immune phenotypes in bladder cancer.

## CONFLICT OF INTEREST

The authors declare no conflict of interest.

## AUTHOR CONTRIBUTIONS

**Hong Weng:** Conceptualization (equal); Data curation (equal); Formal analysis (lead); Investigation (equal); Methodology (lead); Resources (equal); Software (equal); Validation (equal); Visualization (equal); Writing‐original draft (lead); Writing‐review & editing (equal). **Shuai Yuan:** Formal analysis (equal); Investigation (equal); Methodology (equal); Resources (equal); Software (equal); Validation (lead); Visualization (equal); Writing‐original draft (equal); Writing‐review & editing (equal). **Qiao Huang:** Formal analysis (equal); Investigation (equal); Methodology (equal); Resources (equal); Software (equal); Validation (equal); Visualization (lead); Writing‐original draft (equal); Writing‐review & editing (equal). **Xian‐Tao Zeng:** Conceptualization (equal); Data curation (lead); Investigation (equal); Methodology (equal); Project administration (lead); Supervision (equal); Writing‐review & editing (lead). **Xing‐Huan Wang:** Conceptualization (lead); Data curation (lead); Investigation (equal); Project administration (equal); Supervision (lead); Writing‐review & editing (equal).

## Supporting information

Figure S1

Figure S2

Figure S3

Figure S4

Figure S5

Table S1

Table S2

Table S3

Table S4

## Data Availability

All the data were public datasets.
