# Supplementary figures and images for: STAT1 is a key gene in a gene regulatory network related to immune phenotypes in bladder cancer: An integrative analysis of multi‐omics data
Source: J Cell Mol Med. 2021 Feb 19;25(7):3258–71. doi: 10.1111/jcmm.16395 (PMC8034450; doi:10.1111/jcmm.16395)

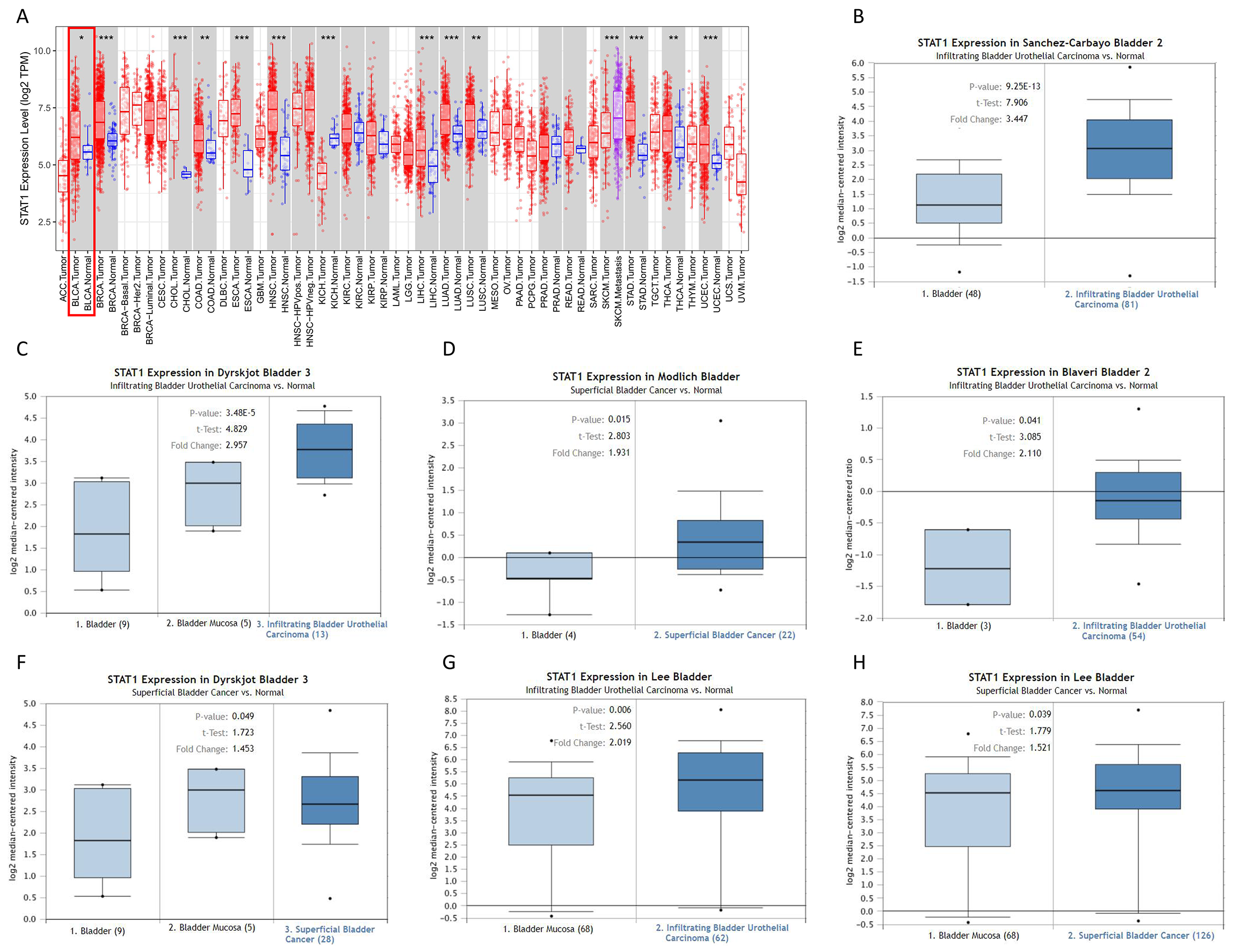

Supplement: Supplementary file 1 — Figure S1 [file JCMM-25-3258-s009.tif]

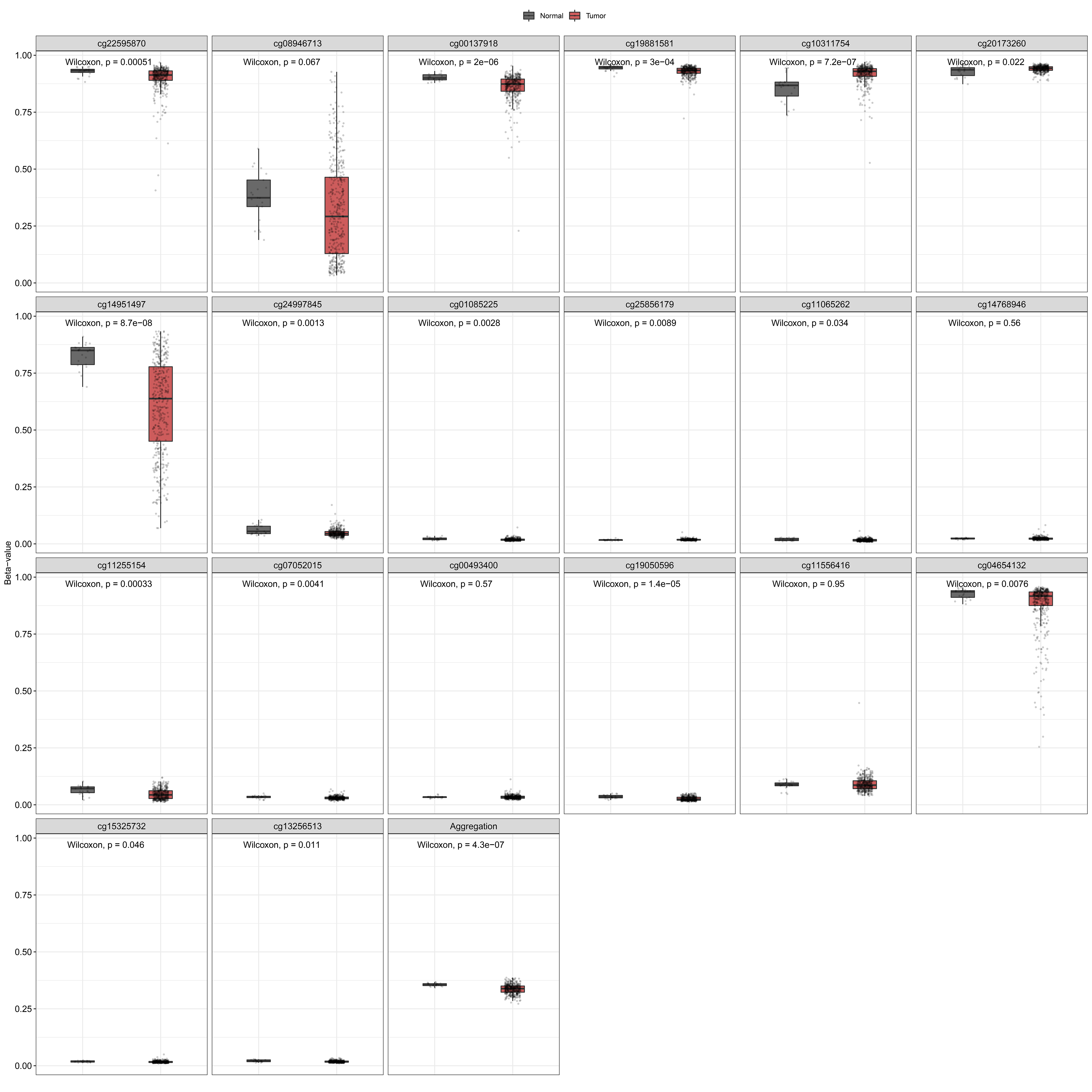

Supplement: Supplementary file 2 — Figure S2 [file JCMM-25-3258-s004.tif]

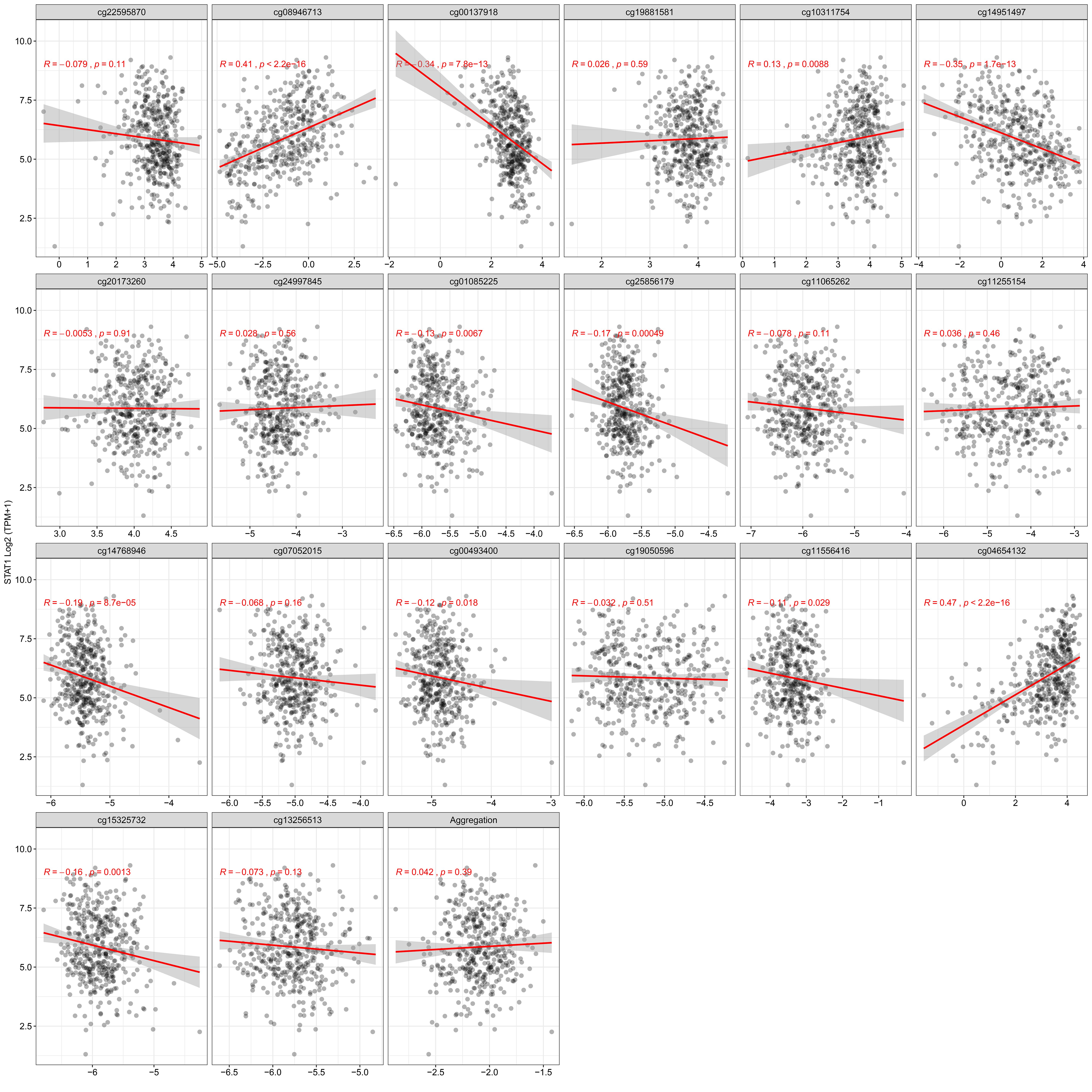

Supplement: Supplementary file 3 — Figure S3 [file JCMM-25-3258-s001.tif]

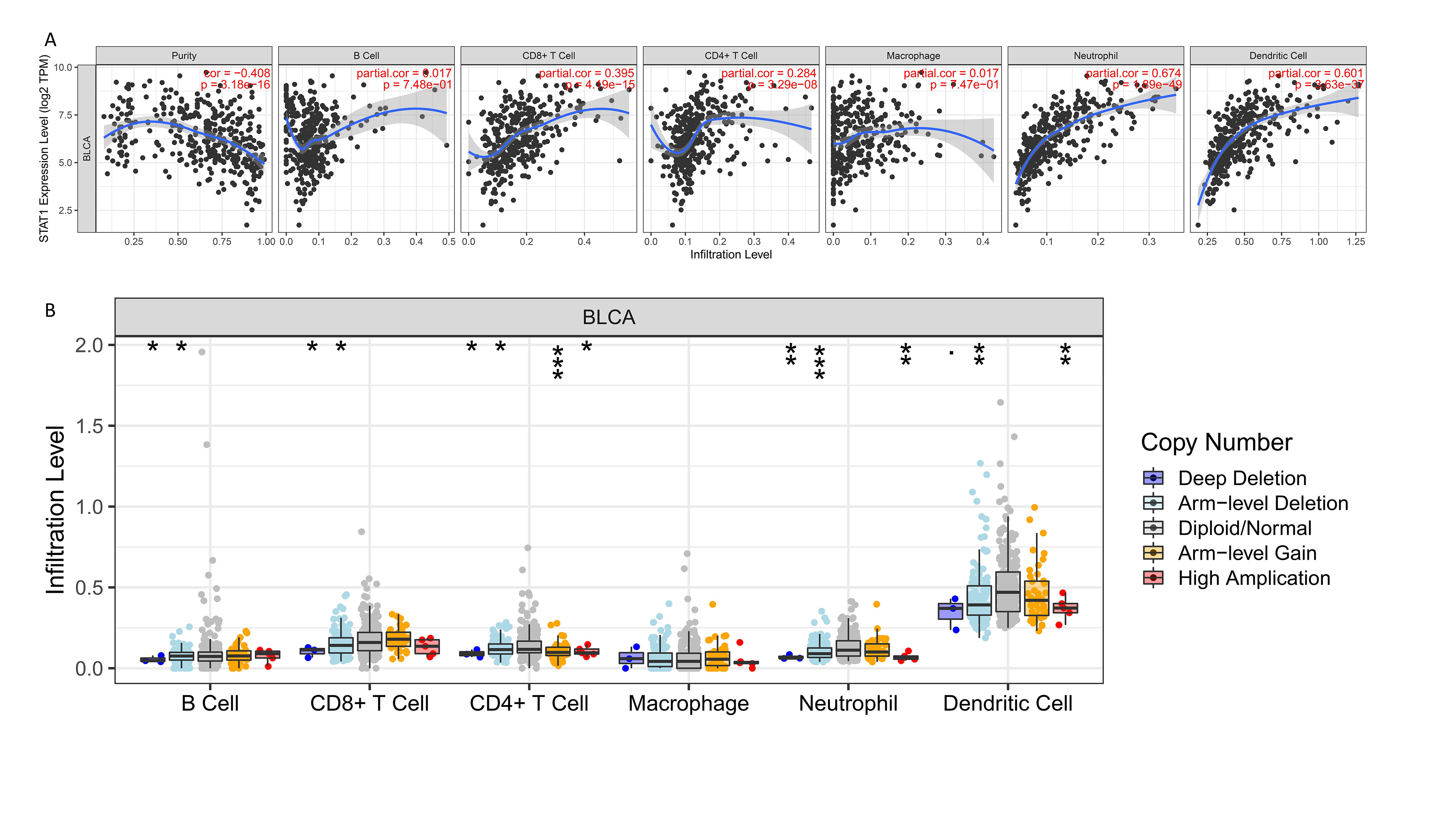

Supplement: Supplementary file 4 — Figure S4 [file JCMM-25-3258-s005.tif]

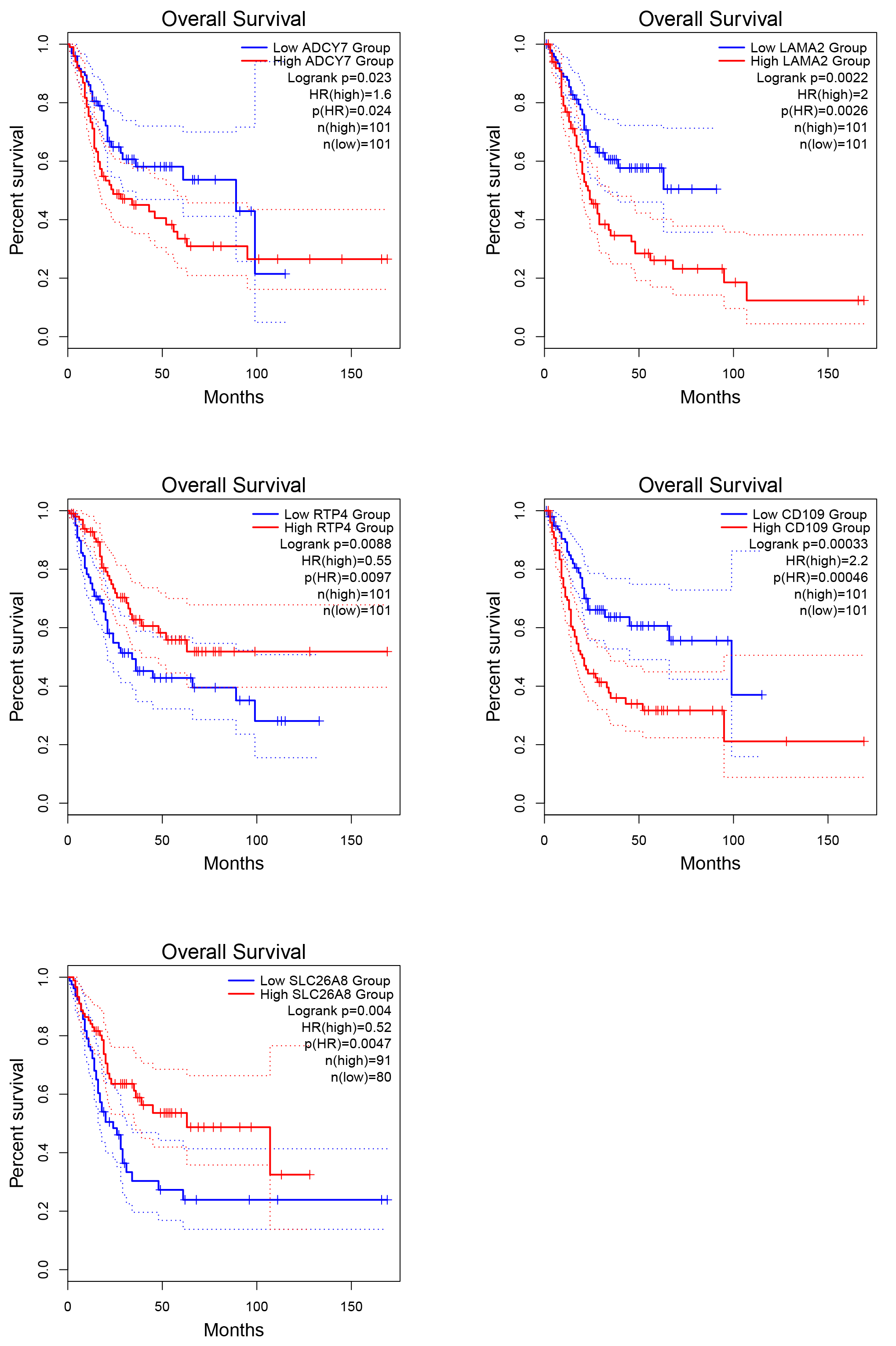

Supplement: Supplementary file 5 — Figure S5 [file JCMM-25-3258-s007.tif]
