# Supplementary material for: STAT1 is a key gene in a gene regulatory network related to immune phenotypes in bladder cancer: An integrative analysis of multi‐omics data
Source: J Cell Mol Med. 2021 Feb 19;25(7):3258–71. doi: 10.1111/jcmm.16395 (PMC8034450; doi:10.1111/jcmm.16395)
Supplement: Supplementary file 6 — Table S1 [file JCMM-25-3258-s002.docx]

**Supplementary Table 1. Clinical information of bladder cancer samples.**

| **Datasets** | **Traits** | **Classification** | **Statistics** |
| --- | --- | --- | --- |
| TCGA-BLCA | Pathological classification (pathologic_T) | TO | 1(0.24%) |
|  |  | T1 | 4(0.97%) |
|  |  | T2 | 119(28.95%) |
|  |  | T3 | 195(47.45%) |
|  |  | T4 | 59(14.36%) |
|  |  | NA | 33(8.03%) |
|  | Gender | Male | 303(73.73%) |
|  |  | Female | 108(26.27%) |
| GSE31684 | Gender | Male | 68(73.12%) |
|  |  | Female | 25(26.88%) |
| GSE13507 | Gender | Male | 135(81.82%) |
|  |  | Female | 30(18.18%) |
