# Supplementary material for: STAT1 is a key gene in a gene regulatory network related to immune phenotypes in bladder cancer: An integrative analysis of multi‐omics data
Source: J Cell Mol Med. 2021 Feb 19;25(7):3258–71. doi: 10.1111/jcmm.16395 (PMC8034450; doi:10.1111/jcmm.16395)
Supplement: Supplementary file 8 — Table S3 [file JCMM-25-3258-s006.docx]

**Supplementary Table 3. Cox regression analysis of methylation probes for overall survival in TCGA-BLCA patients.**

| Probe | Univariate Cox regression | | | |  | Multivariate Cox regression^*^ | | | |
| --- | --- | --- | --- | --- | --- | --- | --- | --- | --- |
|  | HR | 95%CI Lower | 95%CI Upper | p.value |  | HR | 95%CI Lower | 95%CI Upper | p.value |
| cg08946713 | 1.1144 | 1.0224 | 1.2148 | 0.0138 |  | 1.0721 | 0.9710 | 1.1839 | 0.1684 |
| cg00137918 | 0.9444 | 0.7308 | 1.2203 | 0.6616 |  | 0.7603 | 0.5595 | 1.0332 | 0.0799 |
| cg14951497 | 1.1475 | 1.0374 | 1.2694 | 0.0075 |  | 1.1559 | 1.0274 | 1.3004 | 0.0159 |
| cg01085225 | 1.4832 | 0.9825 | 2.2390 | 0.0606 |  | 1.1934 | 0.5653 | 2.5198 | 0.6428 |
| cg25856179 | 0.9352 | 0.4812 | 1.8173 | 0.8433 |  | 0.5759 | 0.2679 | 1.2378 | 0.1575 |
| cg14768946 | 1.4846 | 0.9213 | 2.3921 | 0.1045 |  | 1.6445 | 0.8761 | 3.0869 | 0.1216 |
| cg00493400 | 1.8022 | 1.1308 | 2.8722 | 0.0133 |  | 1.6439 | 0.9588 | 2.8186 | 0.0708 |
| cg11556416 | 0.8724 | 0.6262 | 1.2154 | 0.4197 |  | 0.7727 | 0.5360 | 1.1141 | 0.1672 |
| cg15325732 | 1.2520 | 0.8050 | 1.9472 | 0.3185 |  | 1.1130 | 0.4912 | 2.5216 | 0.7976 |

HR: hazard ratio; CI: confidence interval.

*Adjusted by age, gender, race, and clinical stage.
