# Supplementary material for: STAT1 is a key gene in a gene regulatory network related to immune phenotypes in bladder cancer: An integrative analysis of multi‐omics data
Source: J Cell Mol Med. 2021 Feb 19;25(7):3258–71. doi: 10.1111/jcmm.16395 (PMC8034450; doi:10.1111/jcmm.16395)
Supplement: Supplementary file 9 — Table S4 [file JCMM-25-3258-s003.docx]

**Supplementary Table 4. Cox regression analysis of methylation probes for disease-free survival in TCGA-BLCA patients.**

| Probe | Univariate Cox analysis | | | |  | Multivariate Cox regression* | | | |
| --- | --- | --- | --- | --- | --- | --- | --- | --- | --- |
|  | HR | 95%CI Lower | 95%CI Upper | p.value |  | HR | 95%CI Lower | 95%CI Upper | p.value |
| cg08946713 | 1.0704 | 0.8700 | 1.3171 | 0.5200 |  | 1.1665 | 0.8703 | 1.5635 | 0.3027 |
| cg00137918 | 0.9050 | 0.4879 | 1.6787 | 0.7515 |  | 0.4744 | 0.2020 | 1.1141 | 0.0869 |
| cg14951497 | 1.2253 | 0.9573 | 1.5682 | 0.1066 |  | 1.4141 | 0.9779 | 2.0447 | 0.0656 |
| cg01085225 | 1.1618 | 0.3840 | 3.5151 | 0.7907 |  | 1.9332 | 0.2627 | 14.2260 | 0.5174 |
| cg25856179 | 1.5453 | 0.3421 | 6.9808 | 0.5716 |  | 2.9885 | 0.4123 | 21.6638 | 0.2787 |
| cg14768946 | 0.9567 | 0.2962 | 3.0906 | 0.9411 |  | 0.7689 | 0.1571 | 3.7622 | 0.7456 |
| cg00493400 | 1.5049 | 0.4156 | 5.4490 | 0.5335 |  | 1.3557 | 0.2502 | 7.3440 | 0.7241 |
| cg11556416 | 0.9416 | 0.4472 | 1.9828 | 0.8742 |  | 0.9663 | 0.4250 | 2.1967 | 0.9347 |
| cg15325732 | 0.9535 | 0.3205 | 2.8365 | 0.9318 |  | 0.7835 | 0.1159 | 5.2989 | 0.8025 |

HR: hazard ratio; CI: confidence interval.

*Adjusted by age, gender, race, and clinical stage.
